# Prevalence of mutations in *BRCA* and homologous recombination repair genes and real-world standard of care of Asian patients with HER2-negative metastatic breast cancer starting first-line systemic cytotoxic chemotherapy: subgroup analysis of the global BREAKOUT study

**DOI:** 10.1007/s12282-021-01283-4

**Published:** 2021-08-31

**Authors:** Su-Jin Koh, Shozo Ohsumi, Masato Takahashi, Eisuke Fukuma, Kyung Hae Jung, Takanori Ishida, Ming-Shen Dai, Chuan-Hsun Chang, Tapashi Dalvi, Graham Walker, James Bennett, Joyce O’Shaughnessy, Judith Balmaña

**Affiliations:** 1 Department of Hematology and Oncology, Ulsan University Hospital, Bangeojinsunhwando-ro, Dong-gu, Ulsan, 877 Korea; 2 Department of Breast Oncology, NHO Shikoku Cancer Center, Ehime, Japan; 3 Department of Breast Surgery, NHO Hokkaido Cancer Center, Hokkaido, Japan; 4 Breast Center, Kameda Medical Center, Chiba, Japan; 5 Asan Medical Center, University of Ulsan College of Medicine, Seoul, Korea; 6 Department of Breast and Endocrine Surgical Oncology, Tohoku University Hospital, Miyagi, Japan; 7 Tri-Service General Hospital, Taipei, Taiwan; 8 Cheng Hsin General Hospital, Taipei, Taiwan; 9 AstraZeneca Pharmaceuticals, Gaithersburg, USA; 10 AstraZeneca, Cambridge, UK; 11 Baylor Charles A. Sammons Cancer Center, Texas, USA; 12 Hospital Universitari Vall d’Hebron, Barcelona, Cataluña Spain

**Keywords:** HER2-negative metastatic breast cancer, *BRCA*, Homologous recombination repair, Germline mutations, Somatic mutations

## Abstract

**Background:**

The multinational BREAKOUT study (NCT03078036) sought to determine the prevalence of germline *BRCA1/2* (g*BRCA1/2*) and somatic *BRCA1/2* (s*BRCA1/2*) mutations and mutations in other homologous recombination repair (HRR) genes in women with HER2-negative metastatic breast cancer (MBC) starting first-line chemotherapy.

**Methods:**

Genetic testing for g*BRCA*, s*BRCA*, and HRR gene mutations was performed in patients who started first-line chemotherapy for MBC in the last 90 days (341 patients across 14 countries) who were not selected based on risk factors for g*BRCA* mutations. We report data from the Asian cohort, which included patients in Japan (7 sites), South Korea (10 sites), and Taiwan (8 sites).

**Results:**

Of 116 patients screened, 104 patients were enrolled in the Asian cohort. The median age was 53.0 (range 25–87) years. g*BRCA1/2*, g*BRCA1*, and g*BRCA2* mutations were detected in 10.6% (11/104), 5.8% (6/104), and 4.8% (5/104) of patients, respectively; none had mutations in both g*BRCA1* and g*BRCA2*. g*BRCA1/2* mutations were detected in 10.0% (6/60) and 11.6% (5/43) of patients with hormone receptor-positive and triple-negative MBC, respectively. HRR gene mutations were tested in 48 patients without g*BRCA* mutations, and 5 (10.4%) had at least one HRR mutation in s*BRCA*, *ATM*, *PALB2*, and *CHEK2*.

**Conclusion:**

We report for the first time the prevalence of g*BRCA* and HRR mutations in an Asian cohort of patients with HER2-negative MBC. Our results suggest that *BRCA* mutation testing is valuable to determine appropriate treatment options for patients with hormone receptor-positive or triple-negative MBC.

**Study registration:**

NCT03078036.

**Supplementary Information:**

The online version contains supplementary material available at 10.1007/s12282-021-01283-4.

## Introduction

Breast cancer is one of the most common types of cancer, accounting for up to one-quarter of all cancers in women, with an age-standardized rate of 39.2 cases/100,000 people in East Asian countries [1]. Germline mutations causing functional deficiency in BRCA1/2 (g*BRCA1/2*) are found in about 5% of unselected patients with breast cancer [2, 3]. Approximately 5–10% of breast cancer cases are hereditary, and *BRCA1*/*2* mutations are present in up to 30% of patients with hereditary breast cancers [4]. Functional defects in *BRCA1* and *BRCA2* are also found in approximately 4.2% of unselected Japanese patients with breast cancer [5]. In addition to increased risk of breast cancer, g*BRCA1/2* mutations are associated with substantially increased risk of ovarian, prostate, and pancreatic cancer, and trends suggesting increased risk of melanoma and leukemia [6].

The homologous recombination repair (HRR) pathway is a high-fidelity pathway responsible for repairing double-strand breaks in DNA, and abnormal activity of these proteins may contribute to the development of breast cancer [7, 8]. Therefore, drugs targeting this pathway have been developed as a novel strategy for treating breast cancer in patients with *BRCA1/2* mutations. These include olaparib, a poly (ADP-ribose) polymerase (PARP) inhibitor, that was recently approved for human epidermal growth factor receptor 2 (HER2)-negative, *BRCA1/2* mutation-positive, metastatic breast cancer (MBC) following the results of the OlympiAD study (NCT02000622) [9].

The OlympiAD study compared the efficacy and safety of olaparib versus chemotherapy of the physician’s choice in patients with g*BRCA* mutation-positive, HER2-negative MBC [9]. Although olaparib did not significantly extend overall survival (OS; OlympiAD was not powered to detect a difference in OS between treatment groups), a meaningful benefit on OS was seen in patients who had not previously received chemotherapy for metastatic disease. Subsequent studies have also demonstrated the efficacy of olaparib in patients with mutations in other HRR genes, including prostate cancer [10], and in patients with pancreatic cancer with mutations in g*BRCA1/2* [11].

Genetic testing is an important component of personalized medicine but there are limited data on the prevalence of g*BRCA1/2* mutations in patients treated in real-world settings. Furthermore, *BRCA* mutation testing is usually limited to patients who satisfy the conditions for hereditary breast and ovarian cancer, which may introduce some bias in retrospective studies. Accordingly, the BREAKOUT study was performed to investigate the prevalence of known or suspected deleterious g*BRCA* mutations in prospectively enrolled patients with HER2-negative MBC [12]. Patients were enrolled in real-world settings, regardless of the presence of risk factors for *BRCA* mutations. These data will help estimate the potential population of patients who may benefit from PARP inhibitors.

The secondary and exploratory objectives of the BREAKOUT study were to determine the prevalence of somatic *BRCA* (s*BRCA*) mutations and mutations in other HRR genes, along with the general patient characteristics and first-line treatments for MBC [12].

The BREAKOUT study was performed in real-world settings in 14 countries worldwide, with a primary objective of estimating the prevalence of g*BRCA* mutations among patients with HER2-negative MBC [12]. Here, we report a subgroup analysis of the patients enrolled in three countries in Asia (Japan, South Korea, and Taiwan). Although the study design included a longitudinal follow-up of patients to assess progression-free survival and OS, patient enrollment was terminated in April 2018 and cross-sectional analyses of baseline characteristics and the prevalence of gene mutations were performed.

## Methods

### Ethics

The study adhered to the Declaration of Helsinki, Good Clinical Practice, and Good Pharmacoepidemiology Practice, as well as relevant guidelines in each participating country. The study was approved by ethics committees/institutional review boards at all participating sites and it was registered on ClinicalTrials.gov (NCT03078036).

### Patients

Women with histologically or cytologically confirmed HER2-negative breast cancer with evidence of metastasis who started first-line systemic cytotoxic chemotherapy (not hormonal therapy) for metastatic disease within the last 90 days and who were considered to have exhausted hormone therapy options (if hormone receptor [HR]-positive) were eligible for this study. The major exclusion criteria were current participation in a clinical trial of an investigational oncology drug and current/prior treatment with a PARP inhibitor. Patients provided written informed consent for their medical records to be used in this study, blood sampling to assess g*BRCA* status (if unavailable in medical records), and tumor specimen testing in g*BRCA*-negative patients (if sufficient quality and quantity of archival sample was available). To minimize bias, patients were selected regardless of their demographic characteristics, known risk factors for g*BRCA* mutations, or previously recorded g*BRCA* mutations.

### Study design

The study was performed in 14 countries (Australia, Bulgaria, Canada, Hungary, Italy, Japan, Poland, Russia, South Korea, Spain, Taiwan, Turkey, United Kingdom, and United States). The sites in Japan, South Korea, and Taiwan are listed in the Online Resource—List of participating institutions. The study sites were selected based on their willingness to participate in the study and were asked to enroll sequential patients with HER2-negative MBC.

**Fig. 1:**
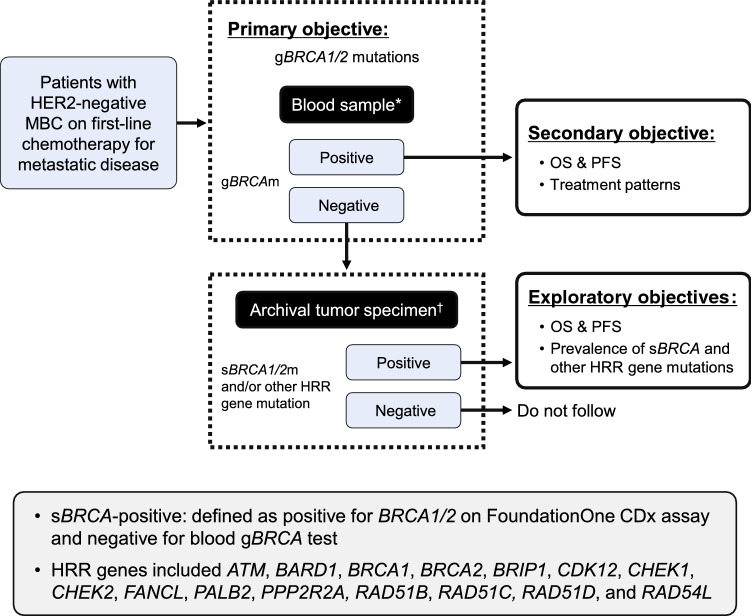
Study design. Modified (restructured figure) from Fig. 1 in O’Shaughnessy et al. [12]. Prevalence of germline BRCA mutations in HER2-negative metastatic breast cancer: global results from the real-world, observational BREAKOUT study. Breast Cancer Research 2020;22:114. Available under a Creative Commons Attribution 4.0 International License. PFS and OS were not assessed due to the early termination of the study. *Blood sample: g*BRCA1/2* mutation status was tested using the BRACAnalysis CDx^®^ assay. ^†^Tumor specimen: HRR gene mutations, including s*BRCA1/2* and other genomic alterations, were tested using the FoundationOne CDx assay. *HER2 *human epidermal growth factor receptor 2,* HRR* homologous recombination repair, *MBC* metastatic breast cancer, *OS* overall survival, *PFS* progression-free survival

Here, we report data obtained in the Asian cohort, which included patients enrolled in Japan (7 sites), South Korea (10 sites), and Taiwan (8 sites). The study was performed in a real-world setting and all treatment decisions were at the investigator’s discretion. The design of the study is illustrated in Fig. 1. Briefly, for all eligible patients, blood samples were taken to assess g*BRCA* mutation status (if g*BRCA* mutation status was unavailable in medical records). For a subset of patients negative for g*BRCA* mutations, archival tumor specimens (if available) were sent to a central laboratory to determine the presence of s*BRCA1/2* mutations and mutations in other HRR genes. Patients signed a separate informed consent form for this procedure.

Data from the patient’s medical records were entered into electronic case report forms (eCRFs) by the investigator or another qualified member of staff. Information recorded in the eCRFs included the country/region, date of birth, race, ethnicity, education, menopausal status, original breast cancer diagnosis date, nicotine use, medical history, comorbidities, breast cancer characteristics, and history of treatment before and at the time of diagnosis of MBC. Any existing biomarker test results for g*BRCA* mutations were entered into the eCRFs, but this information was not to be considered by the investigators when enrolling patients to obtain a representative sample. Blood samples were obtained to test for g*BRCA* mutations if this was not previously documented in the patient’s medical records.

### Blood and tissue testing

Blood samples for g*BRCA* testing were processed locally (where possible) or sent to a central laboratory for testing (BRACAnalysis CDx®; Myriad Genetics Inc., Salt Lake City, UT, USA) and storage. Formalin-fixed, paraffin-embedded tissues were preferred, but core needle biopsies, fine-needle aspirates, and effusion cytologies were also used. Results of g*BRCA* tests were classified as positive, negative, or not determined (Online Resource—Supplemental Table 1). Tissue samples were sent to a central laboratory for analysis using the FoundationOne CDx assay (Foundation Medicine Inc., Cambridge, MA, USA [13]) to detect mutations in the following HRR genes: *ATM*, *BARD1*, *BRCA1*, *BRCA2*, *BRIP1*, *CDK12*, *CHEK1*, *CHEK2*, *FANCL*, *PALB2*, *PPP2R2A*, *RAD51B*, *RAD51C*, *RAD51D*, and *RAD54L*. The results of mutation tests performed before baseline were obtained where available.

### Objectives

The primary objective of the study was to determine the prevalence of g*BRCA1/2* mutations, which were classified as described in Online Resource—Supplemental Table 1. For patients who were found to have a g*BRCA* mutation, the planned secondary objectives included the assessment of treatment patterns by line of therapy and prospective evaluation of clinical outcomes, which included progression-free survival and OS. However, due to the limited number of patients enrolled and early termination of the study, analyses of subsequent therapies and clinical outcomes were not possible.

### Statistical analyses

The study was initially designed with cross-sectional and longitudinal components, and it was planned to enroll ~ 2,000 patients. This sample size would have allowed an estimation of the prevalence of g*BRCA* mutations at a precision of ± 2%. Based on the final sample size (*N* = 341), the 95% confidence interval (CI) spanned 6.5% around the primary endpoint (prevalence of g*BRCA* mutations) and 18.4% for the exploratory endpoint prevalence of HRR gene mutations.

Data analyses were conducted using the full analysis set (FAS), defined as all patients who met the eligibility criteria and either had a previous g*BRCA* test or had a blood sample collected for g*BRCA* testing. The analyses of the exploratory endpoints were conducted using an exploratory subgroup, which comprised all patients in the FAS who had been tested for s*BRCA* and/or any HRR gene mutation, including those in whom the genetic status could not be determined.

Data were analyzed descriptively in terms of the number (percent) of patients for categorical variables and as the median (range) for continuous variables. Owing to the exploratory design of the study, no statistical tests were performed to compare the characteristics of patients between those with or without g*BRCA1/2* mutations.

All analyses were performed using SAS version 9.4 (SAS Institute, Cary, NC, USA).

## Results

### Patient disposition

**Fig. 2:**
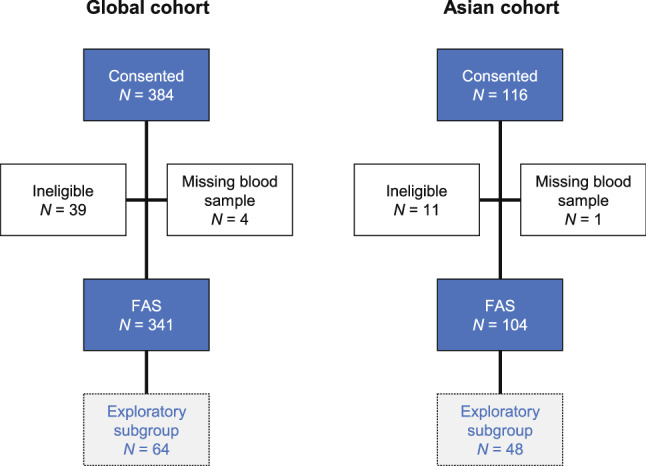
Patient disposition. Data for the global cohort are reprinted from Fig. 2 in O’Shaughnessy et al. [12]. Prevalence of germline BRCA mutations in HER2-negative metastatic breast cancer: global results from the real-world, observational BREAKOUT study. Breast Cancer Research 2020;22:114. Available under a Creative Commons Attribution 4.0 International License. *FAS* full analysis set

The first patient was enrolled on March 13, 2017, and the last patient last visit was June 20, 2018. The database was locked on July 11, 2018. Of 384 patients who were screened and consented to participate, 341 were included in the FAS and 64 in the exploratory subgroup (Fig. 2) [12]. A total of 104 patients were enrolled in the Asian cohort (the focus of this report), of which 45 (43.3%) were from South Korea, 44 (42.3%) were from Japan, and 15 (14.4%) were from Taiwan. The FAS comprised all 104 patients and the exploratory subgroup comprised 48 patients. The g*BRCA* mutation status was assessed prior to baseline in 4 patients (3 patients from South Korea and 1 patient from Japan) and at baseline in 100 patients (42, 43, and 15 patients from South Korea, Japan, and Taiwan, respectively).

### Prevalence of g*BRCA1/2* and s*BRCA1/2* mutations

The primary objective was to determine the prevalence of g*BRCA1/2* mutations. Within the Asian cohort (FAS, *N* = 104), g*BRCA1* and g*BRCA2* mutations were found in 5.8 and 4.8%, respectively (Table 1). As none of the patients had mutations in both genes, the overall prevalence of g*BRCA1* and/or g*BRCA2* mutations was 10.6%. This comprised 7/44 (15.9%) patients from Japan, 3/45 (6.7%) from South Korea, and 1/15 (6.7%) from Taiwan. Mutations in g*BRCA1/2* were found in 11.6% of patients with triple-negative breast cancer (TNBC), all of which were g*BRCA1* mutations. Among patients with HR-positive breast cancer, g*BRCA1/2* mutations were found in 10.0%, which included 1.7% with g*BRCA1* and 8.3% with g*BRCA2* mutations (Table 1).

**Table 1:** Mutation rates in the global and Asian cohorts

	Global cohort	Asian cohort
Full analysis set,^a^ *N*	**341**	**104**
g*BRCA1* only	16 (4.7)	6 (5.8)
g*BRCA2* only	12 (3.5)	5 (4.8)
g*BRCA1* and g*BRCA2*	5 (1.5)	0
g*BRCA1* and/or g*BRCA2*	33 (9.7)	11 (10.6)
TNBC,^b^ *n*	119	43
g*BRCA1* only	9 (7.6)	5 (11.6)
g*BRCA2* only	2 (1.7)	0
g*BRCA1* and g*BRCA2*	0	0
g*BRCA1* and/or g*BRCA2*	11 (9.2)	5 (11.6)
HR-positive,^b^ *n*	215	60
g*BRCA1* only	6 (2.8)	1 (1.7)
g*BRCA2* only	10 (4.7)	5 (8.3)
g*BRCA1* and g*BRCA2*	4 (1.9)	0
g*BRCA1* and/or g*BRCA2*	20 (9.3)	6 (10.0)
Exploratory subgroup,^c^ *n*	**64**	**48**
s*BRCA1* only	1 (1.6)	0
s*BRCA2* only	3 (4.7)	2 (4.2)
s*BRCA1* and s*BRCA2*	0	0
s*BRCA1* and/or s*BRCA2*	4 (6.3)	2 (4.2)
HRR gene mutations other than *BRCA1/2*	5 (7.8)	3 (6.3)

*HR* hormone receptor, *HRR* homologous recombination repair, *TNBC* triple-negative breast cancer

Values presented are *n* (%) unless otherwise stated

^a^Modified (restructured table) from Table 1 in O’Shaughnessy et al. [12]. Prevalence of germline BRCA mutations in HER2-negative metastatic breast cancer: global results from the real-world, observational BREAKOUT study. Breast Cancer Research 22:114. Available under a Creative Commons Attribution 4.0 International License

^b^HR status was unknown in seven patients in the global cohort and one in the Asian cohort

^c^Derived (figure converted to a table) from Fig. 3 in O’Shaughnessy et al. [12]. Prevalence of germline BRCA mutations in HER2-negative metastatic breast cancer: global results from the real-world, observational BREAKOUT study. Breast Cancer Research 2020;22:114. Available under a Creative Commons Attribution 4.0 International License

Bold values indicate the numbers of patients in the full analysis set and exploratory subgroup

The exploratory subgroup comprised 48 patients in whom s*BRCA1/2* mutations and mutations in other HRR genes were assessed. None of these 48 patients had s*BRCA1* mutations, while 4.2% had s*BRCA2* mutations (Table 1). Mutations were also detected in three other HRR genes (*ATM*, *CHEK2*, and *PALB2*) in one patient each (2.1% each; total 6.3%).

### Risk factors for g*BRCA1/2* mutations

The prevalence of g*BRCA1/2* mutations was also assessed in subgroups of patients by family history of breast/ovarian cancer (yes and no) and age at breast cancer diagnosis (≤ 50 years, > 50 years) (Table 2). When analyzed by family history of breast/ovarian cancer, g*BRCA1/2* mutations were found in 40.0% of patients, including 26.7% with g*BRCA1* mutations and 13.3% with g*BRCA2* mutations (versus 5.6, 2.2, and 3.4%, respectively, among patients without a family history of breast/ovarian cancer). Among 57 patients aged ≤ 50 years at breast cancer diagnosis, 14.0% had mutations in either g*BRCA1* (8.8%) or g*BRCA2* (5.3%). Of 44 patients aged > 50 years at breast cancer diagnosis, only one (2.3%) had a mutation in g*BRCA2*, and none had g*BRCA1* mutations. Data for age at breast cancer diagnosis were missing for three patients. Among 81 patients with at least one of TNBC, family history of breast/ovarian cancer, or age ≤ 50 years at breast cancer diagnosis, 11.1% had mutations in either g*BRCA1* (7.4%) or g*BRCA2* (3.7%). A g*BRCA2* mutation was found in 1/21 (4.8%) patients with no risk factors; data were missing for at least one of the risk factors for two patients.

**Table 2:** Mutation rates according to risk factors for g*BRCA* mutations in the Asian cohort (full analysis set)

	*N*	g*BRCA1* only	g*BRCA2* only	g*BRCA1* and/or g*BRCA2*
Overall	104	6 (5.8)	5 (4.8)	11 (10.6)
Family history of breast/ovarian cancer				
Yes, *n*	15	4 (26.7)	2 (13.3)	6 (40.0)
No, *n*	89	2 (2.2)	3 (3.4)	5 (5.6)
Age at breast cancer diagnosis^a^				
≤ 50 years, *n*	57	5 (8.8)	3 (5.3)	8 (14.0)
> 50 years, *n*	44	0	1 (2.3)	1 (2.3)
Any risk factor^b^				
Yes	81	6 (7.4)	3 (3.7)	9 (11.1)
No	21	0	1 (4.8)	1 (4.8)

Values presented are *n* (%).

^a^Age at breast cancer diagnosis was unknown for three patients

^b^At least one of the following: family history of breast/ovarian cancer, age at breast cancer diagnosis ≤ 50 years, or triple-negative breast cancer (data were missing for two patients)

### Characteristics of patients according to g*BRCA1/2* and s*BRCA1/2* status

We assessed the characteristics of patients with mutations in g*BRCA1/2*. Their demographic characteristics are shown in Table 3, disease characteristics and HR status in Table 4, and treatment history in Online Resource—Supplemental Tables 2–4. However, the small sample size of this cohort may preclude meaningful analyses.

**Table 3:** General demographics and family history of cancer in the Asian cohort (full analysis set)

	Overall (*N* = 104)	g*BRCA1/2*m-positive (*N* = 11)	g*BRCA1/2*m-negative (*N* = 93)
Age at enrollment, years	53.0 (25–87)	45.0 (25–54)	55.0 (36–87)
Age at breast cancer diagnosis, years	48.0 (24–86) (*n* = 101)	36.6 (24–51) (*n* = 9)	49.5 (24–86) (*n* = 92)
Post-menopausal at enrollment	73 (70.9) (*n* = 103)	4 (36.4) (*n* = 11)	69 (75.0) (*n* = 92)
Nicotine use, never	85 (85.9) (*n* = 99)	5 (50.0) (*n* = 10)	80 (89.9) (*n* = 89)
ECOG PS^a^			
0	71 (68.3)	9 (81.8)	62 (66.7)
1	26 (25.0)	2 (18.2)	24 (25.8)
2	7 (6.7)	0	7 (7.5)
Family history of breast/ovarian cancer	15 (14.4)	6 (54.5)	9 (9.7)

*ECOG PS* Eastern Cooperative Oncology Group—Performance Status

Values presented are median (range) or *n* (%).

The number of patients with available data is provided where it differs from the overall number of patients. Percentages are based on the number of patients with available data

^a^At initiation of first-line systemic cytotoxic chemotherapy

**Table 4:** Breast cancer characteristics and HR status in the Asian cohort (full analysis set)

	Overall (*N* = 104)	g*BRCA1/2*m-positive (*N* = 11)	g*BRCA1/2*m-negative (*N* = 93)
Time since breast cancer diagnosis, months	33.0 (0.5–357.5) (*n* = 101)	27.1 (3.2–160.6) (*n* = 9)	33.9 (0.5–357.5) (*n* = 92)
T stage at breast cancer diagnosis			
T0 (T0, Tis)	3 (2.9)	1 (9.1)	2 (2.2)
1 (T1, T1a–c)	19 (18.3)	4 (36.4)	15 (16.1)
2 (T2, T2a–c)	54 (51.9)	3 (27.3)	51 (54.8)
3 (T3, T3a–c)	16 (15.4)	2 (18.2)	14 (15.1)
4 (T4, T4a–d)	9 (8.7)	1 (9.1)	8 (8.6)
TX	3 (2.9)	0	3 (3.2)
N stage at breast cancer diagnosis			
N0 (N0, pN0)	37 (35.6)	6 (54.5)	31 (33.3)
N1 (all N1)	36 (34.6)	4 (36.4)	32 (34.4)
N2 (N2, N2a–c)	13 (12.5)	0	13 (14.0)
N3 (N3, N3a–c)	13 (12.5)	1 (9.1)	12 (12.9)
NX	5 (4.8)	0	5 (5.4)
M stage at breast cancer diagnosis			
M0 (all M0)	78 (75.0)	8 (72.7)	70 (75.3)
M1 (all M1)	21 (20.2)	2 (18.2)	19 (20.4)
MX	5 (4.8)	1 (9.1)	4 (4.3)
AJCC stage at breast cancer diagnosis			
0	4 (3.8)	1 (9.1)	3 (3.2)
I	12 (11.5)	2 (18.2)	10 (10.8)
II	42 (40.4)	5 (45.5)	37 (39.8)
III	25 (24.0)	1 (9.1)	24 (25.8)
IV	21 (20.2)	2 (18.2)	19 (20.4)
Histological type at breast cancer diagnosis^a^			
Invasive ductal	83 (79.8)	10 (90.9)	73 (78.5)
Invasive carcinoma NOS	6 (5.8)	0	6 (6.5)
Invasive lobular	5 (4.8)	0	5 (5.4)
Ductal carcinoma in situ	4 (3.8)	1 (9.1)	3 (3.2)
Papillary	1 (1.0)	0	1 (1.1)
Tubular	1 (1.0)	0	1 (1.1)
Other	4 (3.8)	0	4 (4.3)
HR receptor status at enrollment	*n* = 103	*n* = 11	*n* = 92
Estrogen receptor positive	58 (56.3)	5 (45.5)	53 (57.6)
Progesterone receptor positive	40 (38.8)	4 (36.4)	36 (39.1)

*AJCC* American Joint Committee on Cancer, *HR* hormone receptor, *NOS* not otherwise specified

Values presented are median (range) or* n* (%).

The number of patients with available data is provided where it differs from the overall number of patients. Percentages are based on the number of patients with available data

^a^No patients had lobular carcinoma in situ, mucinous, medullary, Paget’s disease of the nipple with/without invasive carcinoma, or inflammatory histological types

As indicated in Table 3, patients with g*BRCA1/2* mutations tended to be younger and had a better Eastern Cooperative Oncology Group Performance Status, and a higher proportion had a family history of breast/ovarian cancer compared with patients without g*BRCA1/2* mutations.

The distribution of American Joint Committee on Cancer (AJCC) stage was similar in the overall Asian cohort and according to g*BRCA1/2* status (Table 4).

The treatment history prior to the diagnosis of MBC was similar between patients with and without g*BRCA1/2* mutations, with over half of patients having received chemotherapy prior to metastatic disease and a median of 4 cycles of treatment (Online Resource—Supplemental Table 2). The treatments received during metastatic disease prior to first-line chemotherapy were also broadly comparable between the two groups of patients (Online Resource—Supplemental Table 3), with letrozole, bevacizumab, exemestane, fulvestrant, and everolimus being the most common non-chemotherapeutic agents. In terms of first-line cytotoxic chemotherapies for MBC, a greater proportion of patients with g*BRCA1/2* mutations had received two or more unique therapeutic agents compared with patients without g*BRCA1/2* mutations. Paclitaxel and bevacizumab were more frequently used in patients with g*BRCA1/2* mutations (Online Resource—Supplemental Table 4).

The two patients with s*BRCA1/2* mutations were aged 57.0 and 66.0 years at enrollment, without family history of breast/ovarian cancer. The histological type was invasive ductal in both patients, the disease stage was IIA in one patient and III in the other. Both patients were estrogen receptor positive, and one was progesterone receptor positive. One patient had received tamoxifen prior to diagnosis of MBC, and both were treated with paclitaxel as first-line treatment for MBC.

## Discussion

In the Asian cohort of BREAKOUT, a cross-sectional study of patients with HER2-negative MBC, mutations in g*BRCA1/2* were detected in 10.6% of patients in the full analysis set, which included 5.8% with g*BRCA1* mutations and 4.8% with g*BRCA2* mutations. Screening for g*BRCA1/2* mutations is now an important aspect of the diagnosis and management of breast cancer considering the changing treatment landscape after the recent approval of PARP inhibitors, such as olaparib [14]. The findings obtained in the Asian cohort generally reflect those obtained in the overall cohort (*N* = 341), where 9.7% of patients had mutations in g*BRCA1/2* [12].

Significant variability in the prevalence of g*BRCA1/2* mutations was reported in prior studies of unselected patients with breast cancer [15–18], which may represent variability among ethnic groups and geographical areas, or other clinical factors [19–22]. Prior to the BREAKOUT study, no studies had examined the prevalence of g*BRCA1/2* mutations within a global population of patients with HER2-negative MBC who were not selected based on risk factors for g*BRCA* mutations.

Another clinically relevant finding of our study is that the prevalence of g*BRCA1/2* mutations was similar between patients with TNBC (11.6%) or HR-positive breast cancer (10.0%). In a study in South Korea involving 1628 unselected women with TNBC (999 underwent molecular testing), 131 (13.1%) had mutations in *BRCA1/2* [23]. The authors also noted that the *BRCA1/2* mutation carriers were younger at breast cancer diagnosis than non-carriers (mean age 45.5 vs 50.3 years, *P* < 0.0001) [23].

Women with a family history of breast or ovarian cancer are more likely to have *BRCA* mutations associated with worse prognosis and warrant risk assessment, genetic testing, and appropriate interventions [24]. In this Asian cohort, we found a high rate of *BRCA1/2* mutations (40.0%) among those with a family history of breast/ovarian cancer compared with 5.6% among patients with no family history. Although the prognosis of these women was not assessed, their outcomes may be worse than those women without g*BRCA1/2* mutations [25] and women without a family history of breast/ovarian cancer [26]. Considering that *BRCA1/2* mutations are also found in patients with no family history (5.6% in the Asian cohort), genetic testing will help to determine appropriate treatment options for these patients. Although National Comprehensive Cancer Network Guidelines advocate genetic testing in patients satisfying certain criteria [27], the current results suggest that some patients with *BRCA1/2* mutations are overlooked based on these criteria. Therefore, widening the criteria for *BRCA* mutation testing or offering mutation testing to all patients with breast cancer might be clinically valuable to improve the detection and treatment of MBC, and this may become a routine procedure with broader use of PARP inhibitors for treating MBC. Better understanding of the mutational profile is also increasing performance of genetic testing in people with high hereditary risk of breast or ovarian cancer. However, the cost of genetic testing is an important factor in screening programs. Recent studies have suggested that population-based genetic testing is more cost-effective than a strategy based on clinical criteria and family history [28, 29]. Although a recent Japanese study of patients with MBC suggested that *BRCA1/2* profiling combined with olaparib treatment provided a minimal incremental benefit versus standard chemotherapy alone [30], other studies have demonstrated cost-effectiveness of routine/mainstream genetic testing for all patients diagnosed with breast cancer to guide subsequent personalized therapy [31, 32].

In addition to *BRCA*, we detected mutations in several HRR genes, including *ATM*, *CHEK2*, and *PALB2* in the Asian cohort. These genes encode ATM serine/threonine kinase, checkpoint kinase 2, and partner and localizer of BRCA2, respectively, and are involved in the detection and response to double-stranded DNA breaks through the HRR pathway. Mutations in these genes have been recognized before now [33], including in a recent case–control study in Japan showing that *BRCA1/2*, *PALB2*, and *TP53* are the major hereditary breast cancer genes in unselected patients [5]. Furthermore, preliminary studies have suggested that cancers showing defects or deficiencies in these genes may respond to PARP inhibitors, such as olaparib [34–36]. Studies examining the use of PARP inhibitors in patients with these or other HRR mutations will help clarify their use in patients with mutations in genes other than *BRCA1/2* [37, 38]. Accordingly, genetic testing of other HRR genes, including those documented in this study, may be beneficial.

Somatic mutations in *BRCA1/2* were detected in two patients (4.2%), similar to the prevalence in the global cohort (6.3%) [12]. In a recent study of Japanese patients, somatic mutations were detected in 27 of 108 patients (29 genes), including *BRCA1* in one patient (0.9%) [39]. In another study of breast cancer patients negative for germline *BRCA1/2, PTEN*, and *TP53* mutations, somatic mutations were predominantly detected in *PIK3CA*, *TP53*, *MAP3K1*, *GATA3*, and *PTEN* genes [40]*.* In a study of patients with MBC, cell-free DNA *BRCA1/2* mutations were detected in 13.5% (29/215) of patients, including nine patients with known germline pathogenic mutations, and the others had novel variants [41]. In a large study of 1,000 patients, pathogenic mutations in *TP53* (337 patients) and *APC* (89 patients) were most common; somatic mutations in *BRCA1* and *BRCA2* were found in three patients each (0.3%) [42]. Overall, these data suggest that somatic mutations in *BRCA1/2* are infrequent, and that genetic testing for somatic mutations should encompass a variety of genes.

Finally, we assessed the general characteristics of this Asian cohort with or without g*BRCA1/2* mutations. Although the number of patients with g*BRCA1/2* mutations was small, we observed some differences. In particular, the patients with g*BRCA1/2* mutations were generally younger at breast cancer diagnosis and often had a family history of breast/ovarian cancer. However, other characteristics were similar, including frequency of HR-positivity and time since diagnosis. Furthermore, there were no clear differences in treatments before or at the time of diagnosis of MBC, with the exception of some potential differences in first-line cytotoxic chemotherapies for MBC. Differing characteristics of patients with MBC and g*BRCA* mutations were also reported in some recent studies in the United States [43, 44]. In particular, patients with g*BRCA1/2* mutations tended to be younger at breast cancer diagnosis and have TNBC, but their treatment pathway was similar to that of patients untested for g*BRCA* mutations [43]. It is also notable that the OS was shorter in patients with g*BRCA1/2* mutations, especially those with g*BRCA1* mutations, highlighting the need for appropriate therapies [44].

In the future, it will be necessary to evaluate the most appropriate treatment options for MBC. For example, the VIOLETTE study (NCT03330847) in patients with mTNBC investigated the use of olaparib as 2/3L therapy, or combining olaparib with other molecular targeted drugs, such as ceralasertib (an ATR inhibitor), as has been proposed for ovarian cancer [45, 46]. Furthermore, data from large-scale registries and biomarker studies, such as the PRAEGNANT registry in Germany (NCT02338167) [47, 48] and the international AURORA initiative (NCT02102165) [49], will provide valuable insight into the identity and prognostic relevance of biomarkers for MBC.

### Limitations

Some limitations of this study deserve mention, particularly its smaller-than-planned sample size, which was due to early termination of the study, and enrollment of sequential patients, which may limit generalizability due to clinical filtering of patients at participating sites. Furthermore, since patients treated with PARP inhibitors (i.e., in clinical trials prior to their clinical approval) were excluded from BREAKOUT, it is possible that this influenced the type of institution participating in the study, as larger centers that are commonly involved in clinical trials may have been unable to participate or may have experienced difficulty registering sufficient numbers of patients. In addition, somatic mutations were not assessed in all patients without g*BRCA1/2* mutations, and we could not confirm whether the mutations in other HRR genes were somatic or not.

## Conclusions

To our knowledge, BREAKOUT was one of the first prospective, global studies to assess the prevalence of g*BRCA* mutations and other HRR gene mutations in patients with HER2-negative MBC. *BRCA* testing may be valuable for all patients with HER2-negative MBC, including TNBC or HR-positive breast cancer. Some patients with HER2-negative breast cancer and mutations in HRR genes, particularly *BRCA*, may benefit from treatment with molecular targeted agents, such as PARP inhibitors. Therefore, it is important to assess the characteristics of patients who may benefit from these agents.

## Supplementary Information

Below is the link to the electronic supplementary material.

Supplementary file1 (PDF 225 KB)

## Data Availability

Data underlying the findings described in this manuscript may be obtained in accordance with AstraZeneca’s data-sharing policy, described at: https://astrazenecagrouptrials.pharmacm.com/ST/Submission/Disclosure.

## References

[1] Bray F, Ferlay J, Soerjomataram I, Siegel RL, Torre LA, Jemal A (2018). Global cancer statistics 2018: GLOBOCAN estimates of incidence and mortality worldwide for 36 cancers in 185 countries. CA Cancer J Clin.

[2] Kurian AW, Gong GD, John EM, Miron A, Felberg A, Phipps AI (2009). Performance of prediction models for BRCA mutation carriage in three racial/ethnic groups: findings from the Northern California Breast Cancer Family Registry. Cancer Epidemiol Biomarkers Prev.

[3] Malone KE, Daling JR, Doody DR, Hsu L, Bernstein L, Coates RJ (2006). Prevalence and predictors of BRCA1 and BRCA2 mutations in a population-based study of breast cancer in white and black American women ages 35 to 64 years. Cancer Res.

[4] Valencia OM, Samuel SE, Viscusi RK, Riall TS, Neumayer LA, Aziz H (2017). The role of genetic testing in patients with breast cancer: a review. JAMA Surg.

[5] Momozawa Y, Iwasaki Y, Parsons MT, Kamatani Y, Takahashi A, Tamura C (2018). Germline pathogenic variants of 11 breast cancer genes in 7,051 Japanese patients and 11,241 controls. Nat Commun.

[6] Mersch J, Jackson MA, Park M, Nebgen D, Peterson SK, Singletary C (2015). Cancers associated with BRCA1 and BRCA2 mutations other than breast and ovarian. Cancer.

[7] Keung MYT, Wu Y, Vadgama JV (2019). PARP inhibitors as a therapeutic agent for homologous recombination deficiency in breast cancers. J Clin Med.

[8] Chartron E, Theillet C, Guiu S, Jacot W (2019). Targeting homologous repair deficiency in breast and ovarian cancers: Biological pathways, preclinical and clinical data. Crit Rev Oncol Hematol.

[9] Robson ME, Tung N, Conte P, Im SA, Senkus E, Xu B (2019). OlympiAD final overall survival and tolerability results: olaparib versus chemotherapy treatment of physician's choice in patients with a germline BRCA mutation and HER2-negative metastatic breast cancer. Ann Oncol.

[10] de Bono J, Mateo J, Fizazi K, Saad F, Shore N, Sandhu S (2020). Olaparib for metastatic castration-resistant prostate cancer. N Engl J Med.

[11] Golan T, Hammel P, Reni M, Van Cutsem E, Macarulla T, Hall MJ (2019). Maintenance olaparib for germline BRCA-mutated metastatic pancreatic cancer. N Engl J Med.

[12] O’Shaughnessy J, Brezden-Masley C, Cazzaniga M, Dalvi T, Walker G, Bennett J (2020). Prevalence of germline BRCA mutations in HER2-negative metastatic breast cancer: global results from the real-world, observational BREAKOUT study. Breast Cancer Res.

[13] Foundation Medicine, Inc. FoundationOne CDxTM Technical Information (RAL-0003-02). Available at: https://assets.ctfassets.net/vhribv12lmne/6Rt6csmCPuaguuqmgi2iY8/2ab201a51f5943efe36a4b420210ad9e/FoundationOne_CDx_Technical_Information.pdf. Accessed 12 Mar 2021.

[14] Griguolo G, Dieci MV, Guarneri V, Conte P (2018). Olaparib for the treatment of breast cancer. Expert Rev Anticancer Ther.

[15] Couch FJ, Hart SN, Sharma P, Toland AE, Wang X, Miron P (2015). Inherited mutations in 17 breast cancer susceptibility genes among a large triple-negative breast cancer cohort unselected for family history of breast cancer. J Clin Oncol.

[16] Robertson L, Hanson H, Seal S, Warren-Perry M, Hughes D, Howell I (2012). BRCA1 testing should be offered to individuals with triple-negative breast cancer diagnosed below 50 years. Br J Cancer.

[17] Rosenberg SM, Ruddy KJ, Tamimi RM, Gelber S, Schapira L, Come S (2016). BRCA1 and BRCA2 mutation testing in young women with breast cancer. JAMA Oncol.

[18] Stevens KN, Vachon CM, Couch FJ (2013). Genetic susceptibility to triple-negative breast cancer. Cancer Res.

[19] Kwong A (2016). Genetic testing for hereditary breast cancer in Asia—moving forward. Chin Clin Oncol.

[20] Mavaddat N, Barrowdale D, Andrulis IL, Domchek SM, Eccles D, Nevanlinna H (2012). Pathology of breast and ovarian cancers among BRCA1 and BRCA2 mutation carriers: results from the Consortium of Investigators of Modifiers of BRCA1/2 (CIMBA). Cancer Epidemiol Biomarkers Prev.

[21] Mehrgou A, Akouchekian M (2016). The importance of BRCA1 and BRCA2 genes mutations in breast cancer development. Med J Islam Repub Iran.

[22] Villarreal-Garza C, Weitzel JN, Llacuachaqui M, Sifuentes E, Magallanes-Hoyos MC, Gallardo L (2015). The prevalence of BRCA1 and BRCA2 mutations among young Mexican women with triple-negative breast cancer. Breast Cancer Res Treat.

[23] Ryu JM, Choi HJ, Kim I, Nam SJ, Kim SW, Yu J (2019). Prevalence and oncologic outcomes of BRCA 1/2 mutations in unselected triple-negative breast cancer patients in Korea. Breast Cancer Res Treat.

[24] Owens DK, Davidson KW, Krist AH, Barry MJ, Cabana M, Caughey AB (2019). Risk assessment, genetic counseling, and genetic testing for BRCA-related cancer: US Preventive Services Task Force recommendation statement. JAMA.

[25] Baretta Z, Mocellin S, Goldin E, Olopade OI, Huo D (2016). Effect of BRCA germline mutations on breast cancer prognosis: a systematic review and meta-analysis. Medicine (Baltimore).

[26] Mori H, Kubo M, Kai M, Velasquez VV, Kurata K, Yamada M (2018). BRCAness combined with a family history of cancer is associated with a poor prognosis for breast cancer patients with a high risk of BRCA mutations. Clin Breast Cancer.

[27] National Comprehensive Cancer Network. NCCN Clinical Practice Guidelines in Oncology. Genetic/Familial High-Risk Assessment: Breast and Ovarian. Version 3.2019. 2019.

[28] Manchanda R, Patel S, Gordeev VS, Antoniou AC, Smith S, Lee A (2018). Cost-effectiveness of population-based BRCA1, BRCA2, RAD51C, RAD51D, BRIP1, PALB2 mutation testing in unselected general population women. J Natl Cancer Inst.

[29] Ficarazzi F, Vecchi M, Ferrari M, Pierotti MA (2021). Towards population-based genetic screenings for breast and ovarian cancer: a comprehensive review from economic evaluations to patient perspectives. Breast.

[30] Saito S, Nakazawa K, Nagahashi M, Ishikawa T, Akazawa K (2019). Cost-effectiveness of BRCA1/2 mutation profiling to target olaparib use in patients with metastatic breast cancer. Per Med.

[31] Sun L, Brentnall A, Patel S, Buist DSM, Bowles EJA, Evans DGR (2019). A cost-effectiveness analysis of multigene testing for all patients with breast cancer. JAMA Oncol.

[32] Kemp Z, Turnbull A, Yost S, Seal S, Mahamdallie S, Poyastro-Pearson E (2019). Evaluation of cancer-based criteria for use in mainstream BRCA1 and BRCA2 genetic testing in patients with breast cancer. JAMA Netw Open.

[33] Nones K, Johnson J, Newell F, Patch AM, Thorne H, Kazakoff SH (2019). Whole-genome sequencing reveals clinically relevant insights into the aetiology of familial breast cancers. Ann Oncol.

[34] Ghiringhelli F, Richard C, Chevrier S, Vegran F, Boidot R (2016). Efficiency of olaparib in colorectal cancer patients with an alteration of the homologous repair protein. World J Gastroenterol.

[35] Horak P, Weischenfeldt J, von Amsberg G, Beyer B, Schutte A, Uhrig S (2019). Response to olaparib in a PALB2 germline mutated prostate cancer and genetic events associated with resistance. Cold Spring Harb Mol Case Stud.

[36] Mateo J, Carreira S, Sandhu S, Miranda S, Mossop H, Perez-Lopez R (2015). DNA-repair defects and olaparib in metastatic prostate cancer. N Engl J Med.

[37] Faraoni I, Graziani G (2018). Role of BRCA mutations in cancer treatment with poly(ADP-ribose) polymerase (PARP) inhibitors. Cancers (Basel).

[38] Ohmoto A, Yachida S (2017). Current status of poly(ADP-ribose) polymerase inhibitors and future directions. Onco Targets Ther.

[39] Kim SJ, Sota Y, Naoi Y, Honma K, Kagara N, Miyake T (2021). Determining homologous recombination deficiency scores with whole exome sequencing and their association with responses to neoadjuvant chemotherapy in breast cancer. Transl Oncol.

[40] Kwong A, Cheuk IW, Shin VY, Ho CY, Au CH, Ho DN (2020). Somatic mutation profiling in BRCA-negative breast and ovarian cancer patients by multigene panel sequencing. Am J Cancer Res.

[41] Vidula N, Dubash T, Lawrence MS, Simoneau A, Niemierko A, Blouch E (2020). Identification of somatically acquired BRCA1/2 mutations by cfDNA analysis in patients with metastatic breast cancer. Clin Cancer Res.

[42] Meric-Bernstam F, Brusco L, Daniels M, Wathoo C, Bailey AM, Strong L (2016). Incidental germline variants in 1000 advanced cancers on a prospective somatic genomic profiling protocol. Ann Oncol.

[43] Dalvi T, Maclachlan S, Briceno J, Bennett J, McLaurin K, Hettle R, et al. Demographic, clinical/disease characteristics, and treatment of patients with germline breast cancer susceptibility gene mutated (gBRCAm) metastatic breast cancer: a CancerLinQ study. San Antionio Breast Cancer Symposium, December 4–8, 2018, San Antonio, TX, USA.

[44] Dalvi T, McLaurin K, Briceno J, Nordstrom B, Bennett J, Hettle R, et al. A real-world evidence study of germline BRCA mutations and survival in HER2-negative breast cancer. San Antionio Breast Cancer Symposium, December 4–8, 2018, San Antonio, TX, USA.

[45] Boussios S, Karihtala P, Moschetta M, Karathanasi A, Sadauskaite A, Rassy E (2019). Combined strategies with poly (ADP-Ribose) polymerase (PARP) inhibitors for the treatment of ovarian cancer: a literature review. Diagnostics (Basel).

[46] Kim H, George E, Ragland R, Rafail S, Zhang R, Krepler C (2017). Targeting the ATR/CHK1 axis with PARP inhibition results in tumor regression in BRCA-mutant ovarian cancer models. Clin Cancer Res.

[47] Lux MP, Nabieva N, Hartkopf AD, Huober J, Volz B, Taran FA (2018). Therapy landscape in patients with metastatic HER2-positive breast cancer: data from the PRAEGNANT real-world breast cancer registry. Cancers (Basel).

[48] Fasching PA, Hu C, Hart S, Hartkopf AD, Taran FA, Janni W (2019). Germline BRCA1 and BRCA2 mutations in patients with HER2-negative metastatic breast cancer (mBC) treated with first-line chemotherapy: data from the German PRAEGNANT registry. J Clin Oncol..

[49] Zardavas D, Maetens M, Irrthum A, Goulioti T, Engelen K, Fumagalli D (2014). The AURORA initiative for metastatic breast cancer. Br J Cancer.

