# Correction to: Prevalence of mutations in *BRCA* and homologous recombination repair genes and real-world standard of care of Asian patients with HER2-negative metastatic breast cancer starting first-line systemic cytotoxic chemotherapy: subgroup analysis of the global BREAKOUT study

**DOI:** 10.1007/s12282-021-01299-w

**Published:** 2021-09-25

**Authors:** Su-Jin Koh, Shozo Ohsumi, Masato Takahashi, Eisuke Fukuma, Kyung Hae Jung, Takanori Ishida, Ming-Shen Dai, Chuan-Hsun Chang, Tapashi Dalvi, Graham Walker, James Bennett, Joyce O’Shaughnessy, Judith Balmaña

**Affiliations:** 1 Department of Hematology and Oncology, Ulsan University Hospital, Bangeojinsunhwando-ro, Dong-gu, Ulsan, 877 Korea; 2 Department of Breast Oncology, NHO Shikoku Cancer Center, Ehime, Japan; 3 Department of Breast Surgery, NHO Hokkaido Cancer Center, Hokkaido, Japan; 4 Breast Center, Kameda Medical Center, Chiba, Japan; 5 Asan Medical Center, University of Ulsan College of Medicine, Seoul, Korea; 6 Department of Breast and Endocrine Surgical Oncology, Tohoku University Hospital, Miyagi, Japan; 7 Tri-Service General Hospital, Taipei, Taiwan; 8 Cheng Hsin General Hospital, Taipei, Taiwan; 9 AstraZeneca Pharmaceuticals, Gaithersburg, USA; 10 AstraZeneca, Cambridge, UK; 11 Baylor Charles A. Sammons Cancer Center, Texas, USA; 12 Hospital Universitari Vall d’Hebron, Barcelona, Cataluña Spain

## Correction to: Breast Cancer 10.1007/s12282-021-01283-4

The article “Prevalence of mutations in *BRCA* and homologous recombination repair genes and real-world standard of care of Asian patients with HER2-negative metastatic breast cancer starting first-line systemic cytotoxic chemotherapy: subgroup analysis of the global BREAKOUT study”, written by Su Jin Koh, Shozo Ohsumi, Masato Takahashi, Eisuke Fukuma, Kyung Hae Jung, Takanori Ishida, Ming Shen Dai, Chuan Hsun Chang, Tapashi Dalvi, Graham Walker, James Bennett, Joyce O’Shaughnessy, Judith Balmaña, was originally published electronically on the publisher’s internet portal on 31 August 2021 without open access. With the author(s)’ decision to opt for Open Choice the copyright of the article changed on 17 September 2021 to © The Author(s) 2021 and the article is forthwith distributed under a Creative Commons Attribution 4.0 International License, which permits use, sharing, adaptation, distribution and reproduction in any medium or format, as long as you give appropriate credit to the original author(s) and the source, provide a link to the Creative Commons licence, and indicate if changes were made. The images or other third party material in this article are included in the article's Creative Commons licence, unless indicated otherwise in a credit line to the material. If material is not included in the article's Creative Commons licence and your intended use is not permitted by statutory regulation or exceeds the permitted use, you will need to obtain permission directly from the copyright holder. To view a copy of this licence, visit http://creativecommons.org/licenses/by/4.0/.

