# Supplementary material for: Prevalence of mutations in BRCA and homologous recombination repair genes and real-world standard of care of Asian patients with HER2-negative metastatic breast cancer starting first-line systemic cytotoxic chemotherapy: subgroup analysis of the global BREAKOUT study
Source: Breast Cancer. 2021 Aug 31;29(1):92–102. doi: 10.1007/s12282-021-01283-4 (PMC8732904; doi:10.1007/s12282-021-01283-4)
Supplement: Supplementary file 1 — Supplementary file1 (PDF 225 KB) [file 12282_2021_1283_MOESM1_ESM.pdf]

Electronic supplementary materials

Breast Cancer

Prevalence of mutations in *BRCA* and homologous recombination repair genes and real-world standard of care of Asian patients with HER2-negative metastatic breast cancer starting first-line systemic cytotoxic chemotherapy: subgroup analysis of the global BREAKOUT study

Su-Jin Koh, Shozo Ohsumi, Masato Takahashi, Eisuke Fukuma, Kyung Hae Jun, Takanori Ishida, Ming-Shen Dai, Chuan-Hsun Chang, Tapashi Dalvi, Graham Walker, James Bennett, Joyce O'Shaughnessy, Judith Balmaña

Please address all correspondence to:
Su-Jin Koh
Department of Hematology and Oncology, Ulsan University Hospital, 877, Bangeojinsunhwando-ro, Dong-gu, Ulsan, Korea
Tel: +82-10-5417-9352
Fax: +82-52-250-8978
E-mail: sujinkoh@uuh.ulsan.kr

List of participating institutions

Country	Institution	Number of consented patients
Japan	Kitano Hospital, The Tazuke Kofukai Medical Research Institute, Osaka	3
Japan	NHO Hokkaido Cancer Center, Sapporo	8
Japan	Tohoku University Hospital, Sendai	6
Japan	St. Luke's International Hospital, Tokyo	4
Japan	NHO Shikoku Cancer Center, Matsuyama	12
Japan	NHO Kyushu Cancer Center, Fukuoka	4
Japan	Tesshokai Kameda General Hospital, Kamogawa	8
S Korea	Ulsan University Hospital, Ulsan	10
S Korea	Asan Medical Center, Seoul	7
S Korea	National Cancer Center, Goyang	5
S Korea	Seoul National University Bundang Hospital, Seongnam	6
S Korea	Severance Hospital, Yonsei University Health System, Seoul	5
S Korea	Seoul National University Hospital, Seoul	3
S Korea	Gangnam Severance Hospital, Yonsei University Health System, Seoul	5
S Korea	CHA Bundang Medical Center, CHA University, Seongnam	3
S Korea	Dong-A University Hospital, Busan	4
S Korea	Chung-Ang University Hospital, Seoul	2
Taiwan	Changhua Christian Hospital, Changhua	1
Taiwan	Chi Mei Medical Center, Tainan	5
Taiwan	China Medical University Hospital, Taichung	1
Taiwan	Kaohsiung Veterans General Hospital, Kaohsiung	3
Taiwan	Tri-Service General Hospital, Taipei	4
Taiwan	National Cheng Kung University Hospital, Tainan	2
Taiwan	E-Da Hospital, Kaohsiung	1
Taiwan	Cheng-Hsin Rehabilitation Medical Center, Taipei	4

Supplemental Tables

Supplemental Table 1 Classification of g*BRCA* mutation results

***BRCA* status**	**Prior *BRCA* test results**	**Baseline g*BRCA* results***
Positive g*BRCA* mutation	*BRCA1*m and/or *BRCA2*m	• Deleterious mutation<br>• Genetic variant, suspected deleterious<br>• g*BRCA* mutation type (classified as g*BRCA1* and/or g*BRCA2*)
No g*BRCA* mutation	*BRCA* wild type	• No deleterious mutation detected<br>• No mutation detected<br>• Genetic variant, favor polymorphism<br>• Genetic variant of uncertain significance<br>• *BRCA* wild type

*If the mutation status could not be determined from the blood sample, the results were classified as not determined g*BRCA* status

Supplemental Table 2 Treatment history prior to diagnosis of metastatic disease in the Asian cohort (full analysis set)

	Overall (*N* = 104)	g*BRCA1/2*m-positive (*N* = 11)	g*BRCA1/2*m-negative (*N* = 93)
Chemotherapy prior to metastatic disease	57 (56.4) (*n* = 101)	7 (63.6) (*n* = 11)	50 (55.6) (*n* = 90)
Time from end of most recent chemotherapeutic agent to enrollment, months	29.5 (1.1–354.6) (*n* = 54)	30.7 (10.7–176.8) (*n* = 7)	28.2 (1.1–354.6) (*n* = 47)
Number of cycles of most recent chemotherapeutic agent	4.0 (1–21) (*n* = 55)	4.0 (3–8) (*n* = 7)	4.0 (1–21) (*n* = 48)
Chemotherapeutic agents[a,b]	*n* = 189	*n* = 27	*n* = 162
Cyclophosphamide	57 (30.2)	8 (29.6)	49 (30.2)
Docetaxel	32 (16.9)	3 (11.1)	29 (17.9)
Doxorubicin	28 (14.8)	3 (11.1)	25 (15.4)
5-fluorouracil	27 (14.3)	5 (18.5)	22 (13.6)
Epirubicin	23 (12.2)	4 (14.8)	19 (11.7)
Non-chemotherapeutic agents prior to metastatic disease	43 (42.2) (*n* = 102)	4 (36.4) (*n* = 11)	39 (42.9) (*n* = 91)
Time from end of most recent non-chemotherapeutic agent to enrollment, months	12.5 (0.9–156.6) (*n* = 38)	3.0 (1.1–156.6) (*n* = 3)	12.6 (0.9–92.8) (*n* = 35)
Non-chemotherapeutic agents[a,b]	*n* = 58	*n* = 4	*n* = 54
Tamoxifen	26 (44.8)	3 (75.0)	23 (42.6)
Letrozole	17 (29.3)	1 (25.0)	16 (29.6)
Anastrozol	7 (12.1)	0	7 (13.0)

Values presented are median (range) or *n* (%).

The number of patients with available data is given where it differs from the overall number of patients. Percentages are based on the number of patients with available data

[a]Patients may have received more than one agent

[b]Agents used in >10% of patients are shown

Supplemental Table 3 Treatments received during metastatic disease prior to first-line chemotherapy in the Asian cohort (full analysis set)

	Overall (N = 104)	g*BRCA1/2*m-positive (N = 11)	g*BRCA1/2*m-negative (N = 93)
Use of a non-chemotherapeutic agent before first-line chemotherapy	42 (40.4)	5 (45.5)	37 (39.8)
Time from end of most recent non-chemotherapeutic agent to enrollment, months	1.0 (−6.9 to 4.6) (n = 29)	0.3 (0.3 to 0.3) (n = 1)	1.1 (−6.9 to 4.6) (n = 28)
Non-chemotherapeutic agent[a,b]	n = 101	n = 14	n = 87
Letrozol	18 (17.8)	1 (7.1)	17 (19.5)
Bevacizumab	15 (14.9)	4 (28.6)	11 (12.6)
Exemestane	14 (13.9)	1 (7.1)	13 (14.9)
Fulvestrant	14 (13.9)	2 (14.3)	12 (13.8)
Everolimus	13 (12.9)	1 (7.1)	12 (13.8)

Values presented are median (range) or *n* (%).

The number of patients with available data is given where it differs from the overall number of patients. Percentages are based on the number of patients with available data

[a]Patients may have received more than one agent

[b]Agents used in >10% of patients are shown

Supplemental Table 4 First-line systemic cytotoxic chemotherapy for metastatic breast cancer in the Asian cohort (full analysis set)

	Overall (N = 104)	g*BRCA1/2*m-positive (N = 11)	g*BRCA1/2*m-negative (N = 93)
Number of unique chemotherapeutic agents taken			
1	60 (57.7)	4 (36.4)	56 (60.2)
2	38 (36.5)	6 (54.5)	32 (34.4)
3	4 (3.8)	0	4 (4.3)
4+	2 (1.9)	1 (9.1)	1 (1.1)
First-line chemotherapeutic agent[a,b]			
Paclitaxel	32 (30.8)	5 (45.5)	27 (29.0)
Capecitabine	24 (23.1)	2 (18.2)	22 (23.7)
Bevacizumab	14 (13.5)	4 (36.4)	10 (10.8)
Cyclophosphamide	13 (12.5)	2 (18.2)	11 (11.8)
Docetaxel	11 (10.6)	2 (18.2)	9 (9.7)

Values presented are median (range) or *n* (%).

The number of patients with available data is given where it differs from the overall number of patients. Percentages are based on the number of patients with available data

[a]Patients may have received more than one agent

[b]Agents used in >10% of patients are shown
